# The Liver Circadian Metabolic Homeostasis Influence by Combining Ketogenic Diet with Exercise

**DOI:** 10.3390/nu16132039

**Published:** 2024-06-27

**Authors:** Wenbo Xu, Zishi Wang, Cuican Zhang, Wenju Yang, Linchao Fan, Hong Sun

**Affiliations:** 1 Centre for Sport Nutrition and Health, School of Physical Education (Main Campus), Zhengzhou University, Zhengzhou 450001, China; x278645818@163.com (W.X.); wzshzzu@163.com (Z.W.);; 2 School of Life Sciences, Zhengzhou University, Zhengzhou 450001, China; 3 National Health Commission Key Laboratory of Birth Defects Prevention, Henan Key Laboratory of Population Defects Prevention, Henan Institute of Reproduction Health Science and Technology, Zhengzhou 450002, China

**Keywords:** circadian rhythm, ketogenic diet, exercise, metabolism, transcriptome

## Abstract

The ketogenic diet (KD) and regular exercise (EX) are both capable of orchestrating circadian metabolism homeostasis during losing weight. However, the combined effects of these two factors on circadian metabolism remain poorly understood. To determine if the combined treatment yields a superimposed physiological phenotype, we measured weight loss, white adipose, the respiratory exchange ratio (RER), heat production, and activity parameters in individual and combined treatment groups. Surprisingly, none of these metrics displayed a cumulative effect when administered in the combined treatment approach. Additionally, we investigated the impact of combination therapy on molecular homeostasis through using high-throughput liver transcriptomic approaches. The results revealed that individual and combined treatments can reprogram the circadian rhythm; yet, the combined group exhibited a minimum quantity of cyclic transcript genes. Noteworthy, the amplitude of 24 h circadian expression genes was not significantly increased in the combination treatment, indicating that the combined approach has non-overlapping effects on maintenance peripheral metabolism homeostasis. This may be due to the liver requiring less ketogenic and gluconeogenic potential during metabolic processes. This research suggests that combined treatment may have adverse effects on the body’s homeostasis and provide crucial insights for the homeostatic health of athletes or individuals who wish to lose weight.

## 1. Background

The homeostasis of the circadian clock is crucial for regulating metabolic, physiological, and behavioral biological processes [1,2]. The mammalian circadian system comprises a multilevel hierarchical network, with a central oscillator pacemaker situated in the suprachiasmatic nuclei (SCN), and peripheral clocks are positioned in almost all tissues, excluding the SCN [3,4]. Environmental cues, such as light change, dietary composition, and physical activity, can trigger modifications in the biological clock. Upon detecting external signal alterations, the master circadian disseminates temporal information to peripheral clocks via autonomic innervation. Furthermore, peripheral clocks are additionally influenced by the local metabolic status of the tissues in which they are located [5].

The generation of molecular circadian clocks entails intricate and cell autonomous feedback loops that operate approximately every 24 h [6]. This process involves the positive limb, comprising the *Clock* and *Bmal1* genes, which form heterodimers and stimulate the expression of the negative limb by binding to E-boxes on crucial target promoters, such as *Cryptochrome* (*Cry1* and *Cry2*) and *Period* (*Per1*, *Per2*, and *Per3*) genes [7]. Another important diurnal loop comprises *RORs* and the repressor *REV-ERBa*/*REVERBb* genes. *REV-ERB* represses the transcription of *Bmal1*, while *RORs* activate it. Notably, disruptions in the circadian clock may lead to alterations in metabolic homeostasis. For instance, the specific deletion of *Clock* genes in the liver can result in hyperglycemia [8]. Similarly, deletion of the *Rev-erbα/β* genes reduces bile acid synthesis and hepatic accumulation [9,10]. Furthermore, the core circadian clock coordinates the daily fluctuations of clock control genes (CCGs), which work together to regulate a diverse array of processes [11], including lipogenesis, fatty acid oxidation, glucose metabolism, and various rate-limiting enzymes in multiple metabolic pathways [2,12]. 

The ketogenic diet (KD), renowned for its high-fat, adequate protein, and low-carbohydrate composition, has proven effective in reducing hunger and inducing metabolic changes [13]. Its application in managing body adiposity and weight has garnered significant attention. However, certain prior studies have observed that mice treated with KD exhibited decreased liver circadian homeostasis during losing weight. Alarmingly, athletes adhering to a KD diet may face a decline in endurance, physical composition, and strength performance, potentially disrupting metabolic homeostasis to a certain extent [14,15,16]. While the synergistic application of KE (KD and exercise: EX) may enhance body mass reduction, the understanding of the metabolic homeostasis changes resulting from this combination remains relatively rudimentary.

To do this, we investigated the impact of individual and combined treatments on physiological phenotypes related to weight loss in mice. Our findings indicate that, even though mice undergoing combined KE treatment displayed the lightest body weight, there was no cumulative effect observed in the tested physiological markers. We further analyzed the daily liver peripheral transcriptome across different treatment groups. Previous studies revealed that 10% of all liver transcriptome mRNAs are expressed in a circadian manner [17], playing crucial roles in gluconeogenesis, de novo lipogenesis, and other metabolic processes [18]. However, our results showed that combined treatment mice exhibited the least circadian expression genes in liver transcriptomes. Moreover, the amplitude of genes implicated in ketogenesis and the metabolic pathways did not demonstrate a prominent superimposition in the combined treatment, which accounted for the non-significant phenotypic results. This research highlights the adverse impact of combined treatment on maintaining molecular circadian rhythm homeostasis, providing novel insights into the body homeostasis of athletes with specialized dietary regimens, which may directly lead to athletic performance decreases.

## 2. Materials and Methods

### 2.1. Animals Treatment

Age-matched male C57BL/6J mice (Beijing HFK Bioscience Co., Ltd., Beijing, China) were maintained under a controlled temperature of 23–25 °C and a 12-h light/12-h dark cycle. After a week of adaptation, mice were blindly randomized into the following four groups: normal diet without exercise (C), ketogenic diet without exercise (KD), normal diet plus exercise (EX), and ketogenic diet plus exercise (KE) for 8 weeks. The normal diet contained 15.8% kcal from fat, 20.3% kcal from proteins, and 63.9% kcal from carbohydrates. The ketogenic diet was based on 89.91% kcal from fat and 9.99% kcal from proteins.

Exercise training was conducted using a motorized running wheel (Jinan Yiyan Technology Development Company, Jinan, China) [13]. The initial exercise protocol started with 10 rotations per minute (r/min) for 20 min on the first day, gradually increasing to 25 min on the second day, 30 min on the third day, then 30 min at 13 r/min on the fourth day and 15 r/min on the fifth day during the first week. In the subsequent weeks (2–8), the mice ran at a fixed speed of 20 r/min for 30 min. The exercise protocol was well tolerated, and the mice exhibited no signs of exhaustion or injury [19]. The mice had access to food and water ad libitum during this experiment, and their body weight was measured weekly. The animal care and use were in accordance with the guidelines of the Animal Care and Use Committee of Zhengzhou University (ZZUIRB 2023-324) and the Guide for the Care and Use of Laboratory Animals of China.

### 2.2. Metablic Cage Date Acqisition

After the 8-week experiment, metabolic cages (Columbus Instruments, CLAMS, Columbus, OH, USA) were utilized to measure several metabolic parameters by indirect calorimetry (*n* = 6 per group). Individually housed mice were acclimated to the respirometry chamber, and the VCO_2_, VO_2_, heat production, and spontaneous wheel activity parameters were recorded every 6 min for a continuous 72 h. The initial 24 h served as a period of acclimation for the mice to the metabolic cage, while the remaining 48 h were included for data analysis. The respiratory exchange ratio (RER) (RER = VCO_2_/VO_2_) was calculated using the Oxymax 5.66 software.

### 2.3. Liver Transcriptome Sequencing

The remaining mice were anesthetized using pentobarbital sodium (3%), and liver samples were collected every 6 h over a 24-h cycle (Zeitgeber time (ZT) 0, ZT6, ZT12, ZT18, and ZT24). After RNA extraction was completed, the Bioanalyzer 2100 (Agilent, Santa Clara, CA, USA) was utilized to test the RNA integrity and confirmed by electrophoresis with denaturing agarose gel. Ultimately, we obtained RNA samples that met the sequencing requirements.

For the subsequent analysis, the RNA was reverse transcribed into cDNA using SuperScript™ II Reverse Transcriptase (Invitrogen SuperScript™ II Reverse Transcriptase, Invotrogen, Carlsbad, CA, USA). Following this, we augmented the reaction mixture with dUTP Solution, RNase H, and DNA Polymerase I to catalyze the synthesis of the second-strand cDNA. The resulting products were then subjected to screening, purification, and digestion procedures to ensure their quality. Afterward, polymerase chain reaction (PCR) was employed to amplify the products, ultimately resulting in a final cDNA library with an average insert size of 300 ± 50 bp. Finally, we executed 2 × 150 bp paired-end sequencing (PE150) on an Illumina Novaseq™ 6000 sequencing platform, procured from LC-Bio Technology Co., Ltd. in Hangzhou, China.

### 2.4. Transcriptome Date Analysis 

Nest, we employed Hisat2 v2.2.1 to compare and analyze the data against the reference genome and used edgeR to calculate the TMM value of the genes. The Jonckheere–Terpstra–Kendall (JTK_CYCLE) based on TMM data was used to determine 24-h circadian expression genes [6,20] and the genes considered as circadian with the Bonferroni-adjusted *p*-value cut-off of 0.01 [21]. Amplitudes of circadian genes in different groups were compared by the *t*-test. At last, the phase- and amplitude-changed gene pathways were clustered based on the Gene Ontology (GO) annotation [22].

### 2.5. Other Statistical Analysis

The physiological data were presented as the mean ± SEM, the rhythmicity of different physiological parameters was analyzed using the cosinor method [23], and one-way or two-way ANOVA with post hoc analysis was used to compare the physiological parameter differences. All tests were considered significant with a *p*-value < 0.05, and * represents *p*-value < 0.05, ** represents *p*-value < 0.01, *** represents *p*-value < 0.001 in different figures. 

## 3. Results

### 3.1. Physiological Phenotype

**Figure 1:**
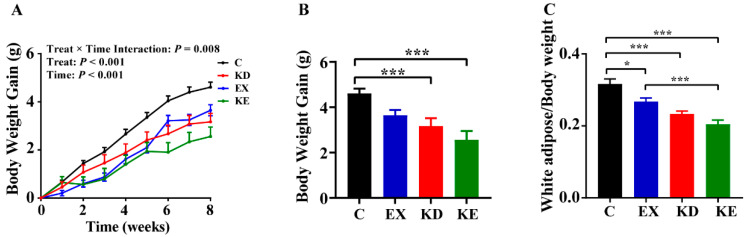
Changes in body weight and white adipose across different treatments. (**A**) Body weight changes from the start of the intervention to the end of the intervention. (**B**) Body weight gain over the 8-week period. (**C**) Percentage of white adipose throughout the 8-week intervention. * *p* < 0.05, *** *p* < 0.001.

To evaluate the effect of individual and combined treatments on weight loss, we monitored the changes in mice body weight and white adipose changes across different treatments. The results showed that weight changes were influenced by treatments and time. As anticipated, the individual and combined treatments all reduced weight gain compared to group C, with the combined treatment mice displaying the lowest body weight (Figure 1A,B). Similarly, the trend was observed in white adipose tissue, where the combined treatment group displayed the maximum decrease (Figure 1C). However, for these parameters, the combined treatment did not exhibit a significant additive effect.

**Figure 2:**
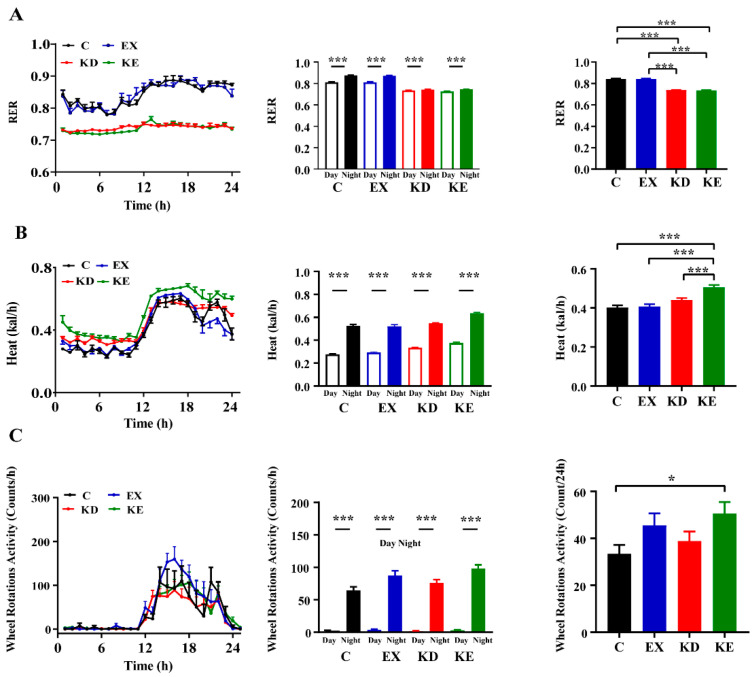
Metabolic parameter changes of different conditions. (**A**) Metabolic parameters of RER. (**B**) Heat production. (**C**) Wheel locomotor activity. Left: The 24-h variation trends of the RER, heat, and activity values. Middle: Differences in the metabolic parameters between the light and dark phases. Right: Average 24-h values of the metabolic parameters. * *p* < 0.05, *** *p* < 0.001.

To identify metabolic state differences resulting from the individual and combined treatments, we utilized indirect calorimetry to analyze the RER, heat, and activity changes of mice. Our analysis revealed that all metabolic parameters exhibited a robust 24-h circadian rhythmicity preference, unaffected by the various treatments (Table S1). Furthermore, the tested metabolic parameters showed significantly elevated phenomenon during the dark phase, attributable to the nocturnal activity characteristics of mice (Figure 2A–C, left and middle). Notably, mice fed regular chow diet (C, KE) exhibited similar RER, while the ketogenic diet-fed groups (KD, KE) maintained consistently lower and flatter RER, suggesting that diet exerts a greater influence on RER than exercise. Finally, we observed that the heat and activity levels were most improved in KE mice, whereas the combined treatment did not perform a significant additive effect, as seen in the weight loss results (Figure 2A–C, right).

### 3.2. Diurnal Transcription Influence by Different Treatments

**Figure 3:**
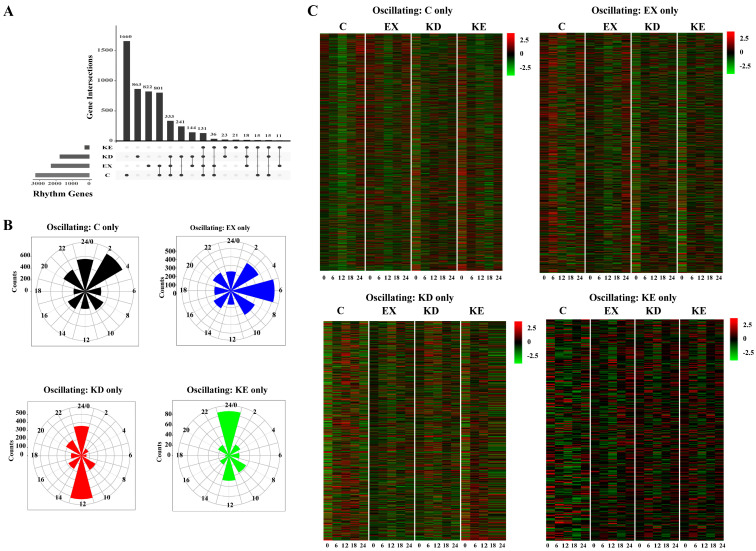
Autonomous transcriptional output of different treatments. (**A**) The number of rhythmic genes under different treatments and combinations. (**B**) Radar plots representing the peak of the diurnal rhythmic transcript in different treatments. (**C**) Heatmaps representing the diurnal rhythmic genes expression patterns in different treatments.

To elucidate the molecular mechanisms responsible for the phenotypic variations, we examined the individual and combined effects on liver diurnal transcriptional homeostasis. Remarkably, our findings revealed that only 270 cyclic transcript genes in the combined KE group, whereas 3232 genes in the C group, exhibited significant robust rhythmicity. Furthermore, 2292 and 1768 genes displayed rhythmic expression patterns in the EX and KD groups, respectively. This suggests that the combined treatment weakened the body homeostasis (Figure 3A). Additionally, the unique rhythmic genes displayed peaks at ZT0 and ZT3 in the C group, ZT3 and ZT6 in the EX group, ZT0 and ZT12 in the KD group, and ZT0 in the KE group (Figure 3B,C).

**Figure 4:**
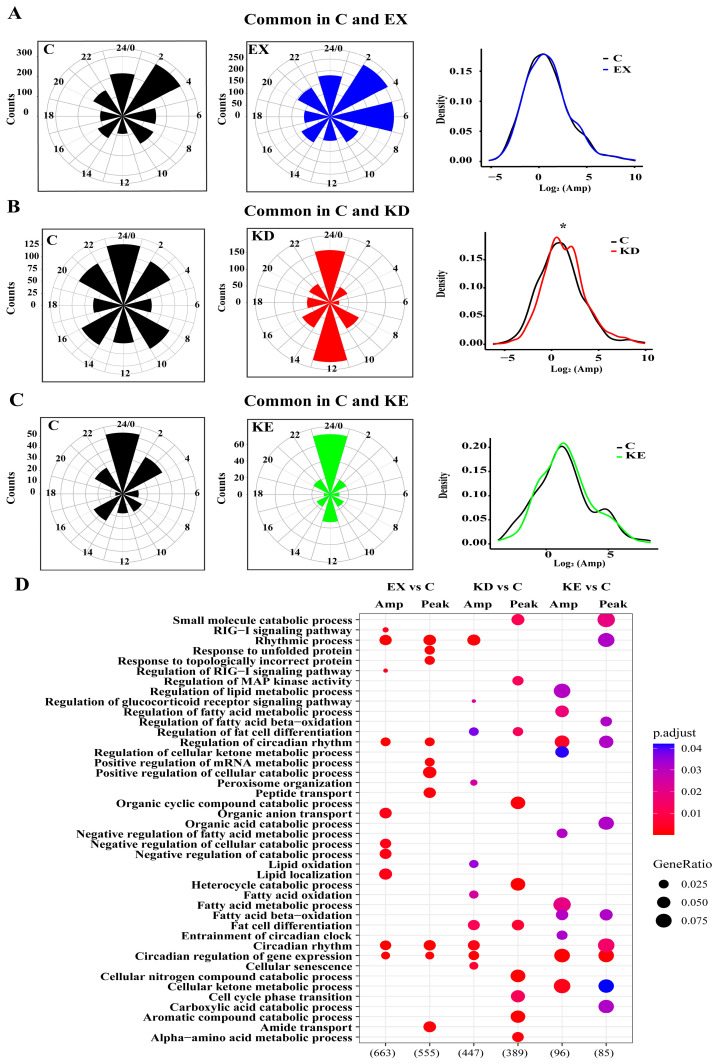
Common autonomous transcriptional output. (**A**) Radar plots and amplitude densities of shared transcript genes in C and EX. (**B**) Radar plots and amplitude densities of shared transcript genes in C and KD. (**C**) Radar plots and amplitude densities of shared transcript genes in C and KE. (**D**) Enrichment functional pathways in GO. * *p* < 0.05.

The common rhythmic genes in different combinations also exhibit different peak and amplitude distributions. We reported that common genes (C, EX) peak at ZT2–ZT7 in EX and ZT2–ZT4 in C, common genes (C, KD) displayed broadened distribution peaks in C and around ZT23–ZT1 in KD, and the common transcripts (C, KE) both exhibited peak phases at ZT23–ZT1. Moreover, the amplitude analysis highlighted that 66.25% of the common oscillator genes (C, KD) manifested a remarkable increase upon KD, while 53.73% common genes (C, EX) showed increased amplitudes in EX. However, only 47.33% of the common genes (C, KE) had an increased amplitude in the KX group (Figure 4A–C). This variation underscores the transcriptional reprogramming that occurs in response to dietary and exercise challenges to liver clocks.

We further delved into the functional enrichment of genes exhibiting amplitude and phase changes. Compared to the normal group, the amplitude overrepresentation-related genes in EX were enriched in the circadian rhythm, lipogenesis, and antioxidant signaling pathways, and the higher amplitude genes in KD were involved in the circadian rhythm, cellular senescence, lipid oxidation, and metabolism. Additionally, the gene sets in KE focused on the circadian regulation, cellular ketone, and lipid metabolic processes. Moreover, we found the peak changes of different treatment oscillation transcriptomes were most enriched in the circadian rhythm and metabolic processes (Figure 4D). Collectively, these findings indicate that the individual and combined treatments influenced the phase and amplitude of the circadian genes, ultimately driving physiological phenotype changes.

### 3.3. Combined Effect for Clock Liver Metabolism Homeostasis

**Figure 5:**
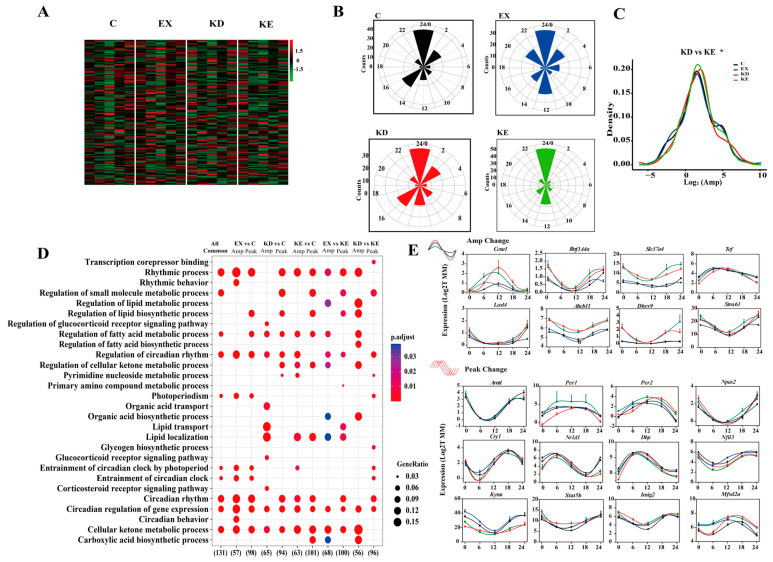
Common autonomous transcriptional output. (**A**) Heatmaps representing the common diurnal rhythmic genes in the four treatments. (**B**) Radar plots representing the phases of common diurnal rhythmic transcripts in the four treatments (**C**) Amplitude density of common transcript genes in the four groups. (**D**) Enrichment functional pathways in GO. (**E**) Different rhythmic gene expression patterns diagram. * *p* < 0.05.

To gain deeper insights into whether the combined treatment had a molecular superposition effect, we conducted a comparative analysis of common rhythmic genes across four distinct groups. Notably, we observed alterations in the core circadian rhythm gene peak changes (Figure 5A,B), while the amplitudes remained relatively stable (Figure 5C,E). This consistency in amplitude was beneficial for the liver’s autonomous homeostasis maintenance. The overlapping gene peaks in the four groups were synchronous at ZT24, and the function analysis of the peak altered the genes primarily involved in the circadian rhythm regulation, lipid metabolic, ketone metabolic, and carboxylic acid biosynthetic processes (Figure 5D). In terms of amplitude, the results indicated that the combined treatment did not exhibit a significant stacking effect, as only KE showed a remarkably higher amplitude combined with KD (Figure 5C). This may clarify the molecular response to lower weight after KE treatments. Intriguingly, the genes that exhibited increased amplitude were involved in fat cell differentiation, fatty acid metabolic and cellular ketone metabolic processes, the carboxylic acid catabolic process, and the cellular response to peptide, which related to weight loss regulation (Figure 5D,E).

## 4. Discussion

Many recent studies have shown that athletes adhering to a KD diet experience a decline in physical composition, potentially disturbing their diurnal metabolic balance. Based on this question, our findings indicated that the combination treatment had no significant stacking effect on weight loss and molecular circadian rhythm homeostasis. Unexpectedly, it even severely impaired the liver’s circadian expression homeostasis, raising concerns about its potential impact on physical health. This discovery could serve as a valuable framework for future investigations into homeostatic health for athletes and weight loss seekers using a KD diet.

The phenotypic performance indicated that, despite having the lowest body mass and white adipose in the combined KE treatments, there was no significant cumulative effect on these parameters. Based on our understanding, KD does not offer superior advantages over non-KD in terms of body fat loss among athletic populations under isocaloric conditions. Additionally, KD can lead to fat-free mass loss in resistance-trained individuals, further explaining the absence of a stacking effect observed in the combination processes [14,24]. Meanwhile, daily RER in the KD-fed groups (KD, KE) was downregulated compared to that of mice fed a normal diet (C, EX). Higher RER values (e.g., 1.0) suggest greater carbohydrate utilization, whereas lower RER values (e.g., 0.7) indicate increased lipid oxidation. The RER reduction could be attributed to the limited carbohydrate supply and lower insulin levels experienced by the KD-fed groups [24,25], as ketogenesis provides an alternative fuel source for the body, conserving glucose. This suggested that KD may play a dominant role in fat mass consumption compared to exercise. Furthermore, the mice treated with the combination approach also demonstrated a non-synergistic effect on the RER, heat, and activity levels, in accordance with past studies that KD does not confer additional benefits in terms of body fat percentage, body mass, VO_2_ max, and aerobic exercise performance among trained participants [26,27]. Notably, we discovered that the circadian rhythm of various metabolic parameters remained unaffected by different treatments, indicating that the diurnal metabolic phenotypic characteristics are not affected by the treatment conditions [17].

**Figure 6:**
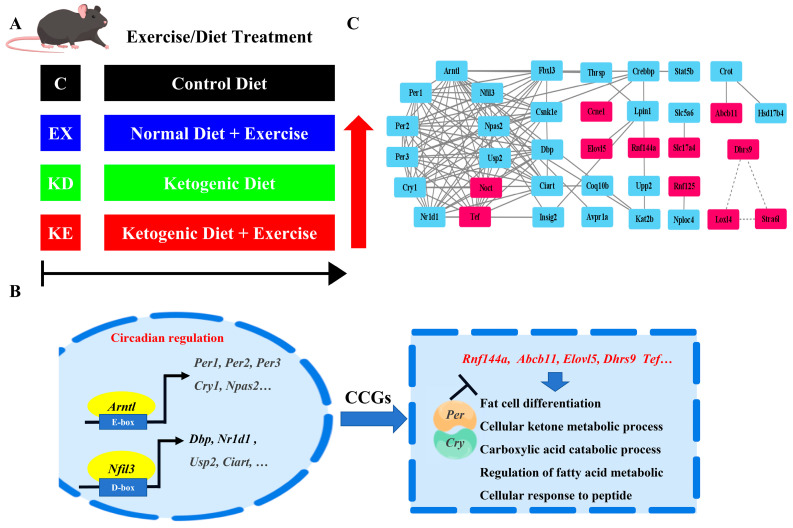
The combined treatment outcome pathway framework for the liver. (**A**) Different treatment groups of C57 for 8 weeks. (**B**) Circadian rhythm and ketone and lipid interaction in the liver. (**C**) Network diagram of higher amplitude genes and the circadian rhythm in the combined processing group.

A 24-h temporal analysis of the liver transcriptome revealed that individual or combined treatments profoundly reprogram oscillating transcripts, altering both the amplitude and peak across different treatment groups. This underscored that the circadian program can be rearranged by diet and exercise [28,29,30]. Importantly, the core clock genes showed a robust cyclic pattern with only minor phase shifts among the groups, which explained the stable maintenance of phenotypic diurnal homeostasis and affirmed the stability of the liver’s fundamental molecular endogenous clock [31]. Nevertheless, the combined KE treatment suppressed a number of rhythmically expressed genes, which may compromise the maintenance of hepatic rhythmic homeostasis and potentially lead to a decline in physical performance and cause various diseases (Figure 6) [32,33]. 

The daily cycle CCGs showed amplitude and peak changes in the lipid and ketogenic metabolic pathways following the individual and combined treatments. For instance, the *Tef*, *Ccnd1*, *Dhrs9*, *Abcb11*, *RNF125*, and *Noct* genes had higher amplitudes in the different treatment groups. These genes play diverse roles, including driving lipid oxidation [34], facilitating adipogenic differentiation while inhibiting preadipocyte proliferation [35], activating lipid metabolism and promoting insulin resistance [36], regulating diet-induced obesity and hypercholesterolemia [37], and decreasing cholesterol and triglyceride synthesis pathways [38],which may play a crucial role in weight loss and lipid reduction [3]. Previous studies have indeed demonstrated that KD can inhibit de novo lipogenesis, accelerate fatty acids, and prompt amino acids to generate ketone bodies. Exercise during weight loss can restructure and activate specific mitochondrial biogenesis, enhance tricarboxylic acid cycle flux, and modulate the lipid oxidation metabolic signaling pathway [1,39]. Coincidentally, the genes in the amplitude changes were involved in these metabolic pathways (Figure 6B,C). However, the amplitude in KE did not exhibit a significant increase, leading us to speculate that this contributed to the non-significant phenotypic results. The peak change genes like *kynu*, which is a transcriptional regulator [40], *Mfsd2a*, which can regulate lipogenesis [41], and *Stat5a*, involved in signal transduction and transcriptional lipid metabolism activation [42], also participate in regulating different metabolic pathways. Additionally, we also found other circadian amplitude regulation properties like *Rnf144a* and *Lox4*, which related to immune responses and promoted immunity. Previous studies have shown that KD and EX are both effective in increasing the antioxidant capacity and oxidative stress, and we speculate that the combined effect may have better antioxidant effects [43].

## 5. Conclusions

Overall, we suggest that the combination treatment has no superior effect on the phenotypes and maintenance of circadian rhythm homeostasis. Our study can provide important implications for the homeostatic health of athletes and individuals who wish to lose weight: that a combination treatment may have adverse effects on the body’s homeostasis. However, our current study was confined to exploring the effects of combined treatment on liver transcriptome metabolism homeostasis, and future research should endeavor to conduct multi-tissue circadian rhythm homeostasis regulation.

## Data Availability

Data are contained within the article and Supplementary Materials.

## Supplementary Materials

The following supporting information can be downloaded at https://www.mdpi.com/article/10.3390/nu16132039/s1: Supplementary Table S1. Circadian rhythm characteristics of the metabolic parameters under different conditions inside the metabolic cages. Supplementary Table S2. Comparison of the results of samples from different groups with the reference genome. Supplementary Table S3. Circadian genes obtained through JTK_CYCLE analysis under different conditions. Supplementary Table S4. GO analysis of the phase- and amplitude-changed circadian genes compared to C. Supplementary Table S5. GO analysis of the phase- and amplitude-changed common circadian genes in all the treatments.
