# Supplementary material for: The Liver Circadian Metabolic Homeostasis Influence by Combining Ketogenic Diet with Exercise
Source: Nutrients. 2024 Jun 27;16(13):2039. doi: 10.3390/nu16132039 (PMC11243221; doi:10.3390/nu16132039)
Supplement: Supplementary file 1 [file nutrients-16-02039-s001.zip › Supplement Table S1.pdf]

Supplement Table S1 Circadian rhythm characteristics of metabolic parameters under different conditions

Treatment	MESOR, M	Amplitude, A	Acrophase, Φ	*P*
**RER**				
C	0.84300	0.0461141100	18.32986996	<0.00001
EX	0.84074	0.048224040	17.46479014	<0.00001
KD	0.73888	0.008679440	15.77843800	<0.00001
KE	0.73552	0.015580270	16.81227763	<0.00001
**Heat**				
C	0.40134	0.177672000	17.90809649	<0.00001
EX	0.40729	0.176086360	17.24004897	<0.00001
KD	0.44106	0.144394080	18.16325184	<0.00001
KE	0.50599	0.180584710	18.30474085	<0.00001
**Wheel**				
C	33.36142	46.59047321	17.4598812	<0.00001
EX	45.50124	68.66954571	16.5101714	<0.00001
KD	38.81002	51.65647158	17.3025468	<0.00001
KE	50.5348	66.28331142	17.7275396	<0.00001
